# Factors associated with knowledge of the postpartum intrauterine contraceptive device and attitude towards its use among women attending antenatal care at Debre Tabor town, Northwest Ethiopia, 2021: a cross-sectional study

**DOI:** 10.1186/s40834-022-00202-y

**Published:** 2023-01-15

**Authors:** Fillorenes Ayalew Sisay, Abeba Belay Ayalew, Besfat Berihun Erega, Wassie Yazie Ferede

**Affiliations:** Department of Midwifery, College of Medicine and Health Sciences, Debre tabor University, Debre Tabor, Ethiopia

**Keywords:** Debretabor, Intrauterine contraceptive device, Knowledge, Pregnancy

## Abstract

**Background:**

Intrauterine contraceptive device is a highly effective, long-acting, reversible family planning method that is safe to use by most postpartum women including those who are breastfeeding. Family planning methods used mainly, the postpartum intrauterine contraceptive device can tackle unintended pregnancy, short birth intervals, and pregnancy-related maternal death. Knowledge and attitude about the postpartum intrauterine contraceptive device were significant predictors of subsequent method use. However, the magnitude, Knowledge, and attitude toward intrauterine contraceptive device is still low in Ethiopia. Nevertheless, limited studies were done to assess Knowledge and attitude toward the postpartum intrauterine contraceptive device and their associated factors. Therefore, this study aimed to assess Knowledge, Attitudes, and associated factors toward postpartum intrauterine contraceptive devices.

**Objective:**

This study aimed to assess Knowledge, Attitudes, and associated factors toward postpartum intrauterine contraceptive devices among pregnant women attending antenatal care at Debre tabor town public health institutions Northwest Ethiopia, 2021.

**Methods:**

an institutional-based cross-sectional study was conducted from March 1- April–30/2021. Four hundred twenty-three participants were recruited by using a systematic random sampling technique. The data were collected through face-to-face interviews using a pretested and structured questionnaire. Multivariable logistic regression analyses were computed to identify factors associated with the outcome variable. An adjusted odds ratio with a 95% confidence interval was computed to determine the level of significance.

**Results:**

Knowledge and attitude towards postpartum intrauterine contraceptive devices were found to be 36 and 48.7% respectively. Government employee (AOR = 4.98,95%CI:2.79–8.91), student (AOR = 5.29,95%CI:1.29–21.80), urban residence (AOR = 1.9095%CI: 1.02–3.53) and ever discussed on a postpartum intrauterine contraceptive device with health care provider (AOR = 6.01,95%CI:3.70–.7.44) were associated with the knowledge about the postpartum intrauterine contraceptive device. Attained secondary education (AOR = 3.22, 95%CI: 1.41–7.31), attended college and above education (AOR = 3.62, 95%CI: 1.75–7.51), government-employee (AOR = 2.76, 95CI:1.11–6.81), student (AOR = 32.10, 95%CI: 3.22–44.79), good knowledge,(AOR = 13.72, 95%CI: 6.63–28.42), ever discussed on a postpartum intrauterine contraceptive device with health care provider (AOR = 2.24,95CI:1.18–4.24), were associated with attitude toward postpartum intrauterine contraceptive device.

**Conclusion and recommendation:**

knowledge and positive attitude toward postpartum intrauterine contraceptive devices were low as compared with other studies. Mothers’ employment status, residence, and discussions about a postpartum intrauterine contraceptive device with healthcare providers improve women’s knowledge about the postpartum intrauterine contraceptive device. Maternal educational status, occupational status, ever discussed postpartum intrauterine contraceptive devices with a health care provider and several antenatal cares follow up were improves women’s attitude towards the postpartum intrauterine contraceptive device. The finding highlights the importance of discussing postpartum intrauterine contraceptive devices during pregnancy, which in turn enhances the knowledge and attitude, of mothers about postpartum intrauterine contraceptive devices.

**Supplementary Information:**

The online version contains supplementary material available at 10.1186/s40834-022-00202-y.

## Introduction

Postpartum IUCD is an intrauterine contraceptive device that is inserted during the postpartum period (up to 48 hours after birth, optimally within10 min of delivery of the placenta [1]. Postpartum Intrauterine contraceptive device (IUCD) is a highly effective, long-acting, reversible family planning method that is safe to use by most postpartum women including those who are breastfeeding [1]. However, there are some contraindications to postpartum Intrauterine contraceptive devices such as active infection, known uterine cavity anomaly, and Ongoing postpartum hemorrhage [2].

The postpartum period was an important time for postpartum IUCD insertion, especially for women who have cultural or geographical limitations to access FP because women were highly motivated for Postpartum family planning and it does not require a return visit [3].

The intrauterine contraceptive device is an inexpensive, non-hormonal, and widely used long-acting reversible modern contraceptive method globally [4, 5]. PPIUCD can promote the health of the mother and the newborn by preventing unwanted and closely spaced pregnancies and their complications [6, 7]. Short inter-birth intervals increased the risk of adverse maternal, infant, and neonatal outcomes such as low birth weight, stillbirth, and maternal and neonatal death [8]. However, Globally, around 25% of live births occur in short intervals, with relatively high proportions in Central Asia (33%) and Sub-Saharan Africa (57%) [9]. In developing countries, more than 100 million women would prefer to avoid pregnancy but they may not be using any form of contraception [10]. This is due to poor knowledge of reliable contraceptive methods, fear of side effects, and inability to return for a contraceptive purpose because of different reasons [11]. Even though copper-bearing IUCD is widely available in Ethiopia and is provided free of charge in government health institutions, it is still underutilized [12]. IUCD use in Ethiopia was only 2%, as reported by EDHS 2016 [12]. World health organization guidelines indicated that improving knowledge about contraception can increase the uptake of any family planning methods [13].

Studies also showed that the major determinate factors for low IUCD utilization were poor knowledge and negative attitude towards IUCD [14, 15]. Even though knowledge and attitude were the determinate factors of IUCD utilization, Knowledge and attitude towards postpartum IUCD were low in the world [16–19]. The study conducted in India indicated that 22.4% of women were aware of PPIUCD and only 37% of women had a positive attitude toward it [16]. Another study done in Uganda showed that 55.9% of women had knowledge about PPIUCD [17]. The study conducted in Ethiopia also showed that only 49% of women had good knowledge and 49.4% of women had positive attitudes toward PPIUCD [18]. Another study conducted in Ethiopia indicated that 43.9% of women had the knowledge and 55% of women had a positive attitude toward PPIUCD [19].

From the above figure, we can conclude that even though, knowledge and attitude were the determinants factors of subsequent use of postpartum IUCD both knowledge and attitude towards PPIUCD was low in the globe as well as in Ethiopia. As far as our knowledge is concerned, few studies were conducted to assess the determinate factors of knowledge and attitude towards postpartum IUCD in Ethiopia since most studies were descriptive. Therefore, this study was conducted to identify factors associated with the knowledge and attitude of women toward PPIUCD.

## Methods

### Study area and period

The study was conducted in Debre tabor town governmental health institutions from March − 1 April–30, 2021. Debre tabor is the capital city of the south Gondar zone, which is about 103 km away from Bahir Dar (the capital city of Amhara regional state) and about 665 Kilometers away from Addis Ababa (the capital city of Ethiopia).

According to the Population projection of Ethiopia for all regions at the woreda level from 2014 to 2017, the total population of the town is estimated to be 96,973 (47,220 females and 49,753 males)) [19].

The town has one specialized Hospital, three Health centers, six private clinics, and six health posts. All public health institutions and three private clinics provide ANC and family planning services.

#### Source and study population

All pregnant women who had antenatal care follow-up at Debre tabor town public health institutions and all pregnant women who had antenatal care follow-up at Debre tabor town public health institutions during the data collection period were the source population and study population respectively.

### Inclusion and exclusion criteria

#### Inclusion criteria

All pregnant women attending antenatal care at Debretabor town public health institutions were included in the study.

#### Exclusion criteria

Women who were seriously ill and were attending ANC at Debretabor town public health institutions during the data collection period were proposed as exclusion criteria but we did not get such participants during the data collection period.

### Sample size determination and procedure

The single population proportion formula was used to calculate the sample size for the first objective by talking a prevalence level of 49% [17]. With the assumption of a 95%, confidence level, and a 5% margin of error.

*n* = (Z ά/2)2 p (1-p)/d2.

*n* = (1.96)2 *0.49(1–0.49)/ (0.05)2 = 384.0.

By adding a 10%, non-response rate the sample size was 422.4 = 423.

The single population proportion formula was used to calculate the sample size for the second objective by talking prevalence level of 49.4%(17).

*n* = (Z ά/2)2 p (1-p)/d2.

*n* = (1.96)2 *0.494(1–0.494)/ (0.05)2 = 384.4.

By adding a 10%, non-response rate the total sample size was 422.84 = 423.

Then the final sample size was found to be 423.

Where:

*n* = the required Sample size.

*p* = prevalence of knowledge about PPIUCD (49% or *P* = 0.49) and prevalence of attitude towards PPIUCD (49.4% or *p* = 0.494).

Z = the value of the standard normal curve score corresponding to the given Confidence interval of 1.96.

d = the permissible Margin of error of 5%.

All health institutions were included in the study and then the number of participants was allocated proportionally to each health institution. The sample was allocated proportionally to all public health institutions based on the monthly average number of women who have ANC follow-up in each institution for the year 2020.

A systematic random sampling technique was used to select study participants and due to the cyclic nature of ANC, cards of the study participants were coded to avoid re-interviewing. The sampling interval k was calculated by dividing the total two months’ source population (1062) by the total sample size (423) which was approximately k = 3. For each of the public health institutions, the constant number K was also calculated and it was the same K = three. This interval was used in all public health institutions to select study participants. The first sample was selected randomly by lottery method among the first three participants (one randomly selected) then every third unit was taken to get the required sample size from each institution.

### Data management and analysis

Data were gathered via face-to-face interviews by using semi-structured and pre-tested questionnaires. A total of four BSc midwives were assigned to collect the data and two MSc midwives were assigned for supervision. Data collectors described the purpose of the study and interview process by emphasizing privacy and confidentiality to the participants before starting the interview process. Data cleaning and crosschecking were done to check for accuracy, completeness, consistencies, and missed values and variables. After the data were checked for completeness and accuracy, coded manually, and then entered into Epi data version 4.6 and exported to Statistical Package of Social Science (SPSS) version 23 for analysis. Both descriptive and analytical statistical procedures were utilized. Binary logistic regression was used to identify factors associated with the outcome variable. A multivariable logistic regression model was fitted to control the possible effect of confounders and finally, the variables, which had an independent association with the outcome variable, were identified based on OR with 95%CI and *p*-value less than 0.05. In the bi-variable logistic regression, the variables associated by COR with *p*-value < 0.2 were entered into the multivariable model. Model fitness was checked using the Hosmer and Lemeshow goodness of fit test.

### Data quality control

The questionnaire was prepared in English, and after that, it was translated to Amharic (the local language) and back to English to maintain the consistency of the tool. To assure the quality of the data, high emphasis was given to designing data collection tools. A pretest was conducted on 5% of the sample size, and necessary corrections were made to the tool accordingly. A one-day training was provided for data collectors and supervisors regarding the objectives of the study, data collection methods, the significance of the study, and the contents of the tool. The data collectors were supervised during data collection, and the collected data were checked for completeness. Both the data collectors and supervisors took on-the-spot corrective measures.

### Operational definition

#### Postpartum intrauterine contraceptive device

An intrauterine contraceptive device that can be inserted post placental, intra cesarean, and within 48 hours of delivery [18].

#### Knowledge of pregnant women about PPIUCD

The knowledge of pregnant women on PPIUCD was measured by the total number of 10 questions with yes/no responses. After computing the mean score of knowledge questions, it was categorized as poor knowledge and good knowledge based on the mean score [20].

#### Good knowledge

women were considered to have good knowledge on PPIUCD if they were answers greater than or equal to the mean score of knowledge questions.

#### Attitude on PPIUCD

The attitude of clients on PPIUCD was measured by using a Likert scale, which assesses whether the clients strongly agree, agree, neutral, disagree, and strongly disagree with the items listed regarding the need for PPIUCD. To measure the attitude it was categorized as a positive attitude and negative attitude based on the mean score.

#### Positive attitude

women who scored above the mean were considered to have a positive attitude towards PPIUCD [21].

## Results

### Socio-demographic characteristics of respondents

A total of 417 pregnant women participated in this study, with a response rate of 98.5%. The respondents’ ages ranged from 18 to 39 years, with a median age of 28 years (IQR, 25 to 31 years). About 44.6% of respondents were in the age range of 25–29 years. Most (89.7%) of the study participants were orthodox Christians. Most of the 394 (94.5%) study participants were married.

More than one quarter (31.4%) of the study participants had completed secondary education. Regarding occupation, less than half (43.9%) of women were housewives. In addition to this, about 25.7% of their husbands were government employees. Nearly three-quarter (72.7%) of women were living in urban areas (Table 1).

**Table 1:** Socio-demographic and socio-economic characteristics of pregnant women attending ANC clinic at Debre tabor town public health institutions, 2021(*n* = 417)

Variable	Frequency	Percentage
Age		
15–24	92	22
25–29	186	44.6
30–34	92	22.1
35–39	47	11.3
Religion		
Orthodox	374	89.7
Muslim	35	8.4
Protestant	8	1.9
Ethnicity		
Amhara	394	94.5
Others(a)	23	5.5
Residence		
Urban	303	72.7
Rural	114	27.3
Educational status		
No education	101	24.2
Primary	62	14.9
Secondary	131	31.4
College and above	123	29.5
Occupational status		
Housewife	183	43.9
Government employee	116	27.8
Private employee	57	13.7
Daily laborer	30	7.2
Farmer	21	5.0
Student	10	2.4
Current marital status		
Married	394	94.5
Divorced	23	5.5
Husband educational status(*n* = 394)		
No education	78	19.8
Primary	95.	24.1
Secondary	100	25.4
College and above	121	30.7
Husband occupational status(*n* = 394)		
Private employee	60	15.2
Daily laborer	55	14
Merchant	91	23.1
Farmer	75	19
Government employee	113	28.7
Household average monthly income		
≤1000	10	2.4
1001–1500	71	17.0
1501–2500	121	29.0
≥2501	215	51.6

Others(a) = Oromo and Tigre

### Reproductive characteristics of the study participants

The median age of women at marriage was 19 with an IQR of 17 to 20 years. More than two-thirds (71.5%) of the study participants had ever given birth, and the median age of women at their first birth was 21.08 (with an IQR of 20–22) years. Among 298 respondents, more than two-thirds (70.1%) of women were multi-parous, and the mean number of alive children was 2.2 (1.263 SD).

Among 298 respondents, about 65.2% of women did not want to have a child within the next two years after delivery. Among those, the majority (91.5%) of women were there for spacing purposes. A bit higher than two-fifth (42.2%) of women discussed PPIUCD with health care providers, and with more than half (51.3%) of respondents, the number of children was decided by both the mother and her husband (Tables 2 and 3).

**Table 2:** Reproductive characteristics of pregnant women attending antenatal care at Debre tabor town public health institutions, 2021(*n* = 417)

Variable	Frequency	Percentage
Age at first marriage		
< 18	97	23.3
≥18	320	76.7
Ever give birth		
Yes	298	71.5
No	119	28.5
Total number of delivery (*n* = 298)		
Primi-para	80	26.9
Multi-para	209	70.1
Grand multi-para	9	3
Age at first child(*n* = 298)		
< 20	88	29.5
≥20	210	70.5
Number of alive children (*n* = 298)		
< 5	298	98
≥5	6	2
More children needed (*n* = 298)		
0	18	6
1	55	18.5
2	108	36.2
3	83	27.9
≥4	34	11.4
Wanted to have a child within two years of delivery		
Yes	145	34.8
No	272	65.2
Reasons for did not want to have a child within two years of delivery (*n* = 272)		
Space	249	91.5
Limit	23	8.5
Number of children wanted to have in life		
< 5	360	86.3
≥5	57	13.7
Discuss about family planning methods with a partner		
Yes	275	65.9
No	142	34.1
Discuss about PPIUD with a health care provider		
Yes	176	42.2
No	241	57.8
Decision on the number of children wanted to have		
Husband	122	29.3
Wife	75	18
Both	214	51.3
God	6	1.4
Responsible person for health care decision		
Husband	135	32.4
Wife	55	13.2
Both	277	54.4
Decision on use of family planning methods		
Wife	19	4.6
husband	147	35.2
both	251	60.2
Ever use modern FP methods		
Yes	301	72
No	116	28
Duration of FP used(*n* = 301)		
< 1 year	106	35.2
≥1 year	195	64.8
Ever shifted FP method(*n* = 301)		
Yes	84	27.9
No	217	72.1

**Table 3:** Knowledge about PPIUCD among pregnant women attending ANC at Debre tabor town public health institutions, North West, Ethiopia, 2021(*n* = 417)

Variable	Category	Frequency	Percent
PPIUD can prevent unwanted pregnancies for more than 10 years	Yes No	217 200	52 48
PPIUCD has no risk of getting sexually transmitted infections	Yes No	174 243	58.3 41.7
PPUCD has no interference with sexual intercourse	Yes No	196 221	47 53
PPIUCD is immediately reversible	Yes No	150 267	36 64
PPUCD cannot cause cervical cancer.	Yes No	133 284	31.9 68.1
PPIUD can be used by breastfeeding mothers	Yes No	190 227	45.6 54.4
PPIUD may cause changes in bleeding pattern	Yes No	192 225	46 54
IUD can be used by HIV positive patients doing well on treatment	Yes No	167 250	40 60
PP IUD is inserted free of charge in Ethiopia	Yes. No	287 130	68.8 31.2
PPIUD can be removed at any time you wish	Yes No	221 196	53 47

### Awareness of pregnant women about PPIUD

**Fig. 1:**
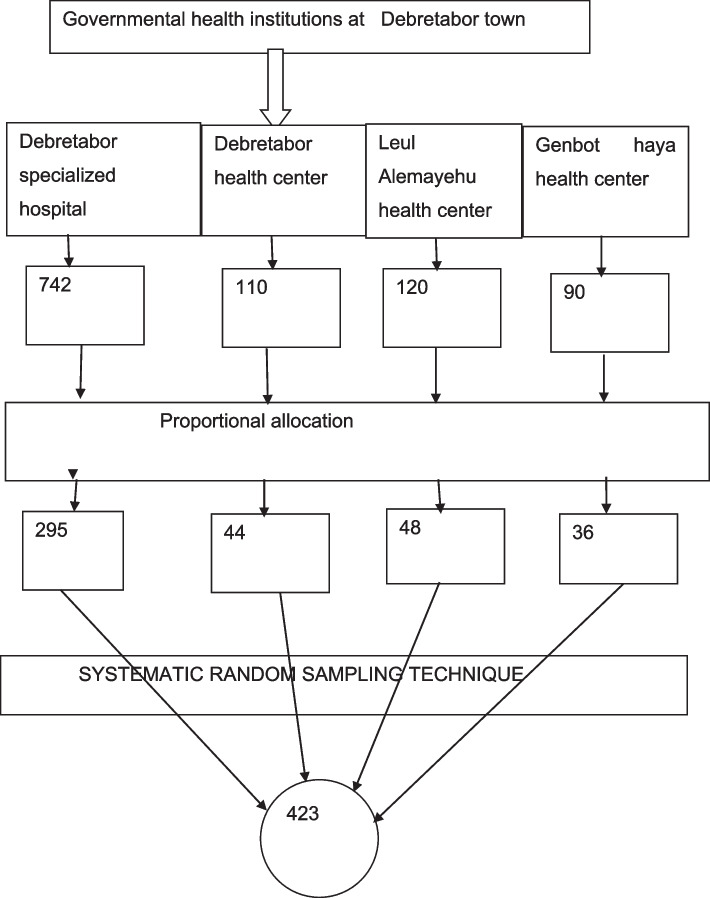
Sampling procedures for the study Factors associated with knowledge of the postpartum intrauterine contraceptive device and attitude towards its use among women attending antenatal care at Debre tabor town, Northwest Ethiopia, 2021

**Fig. 2:**
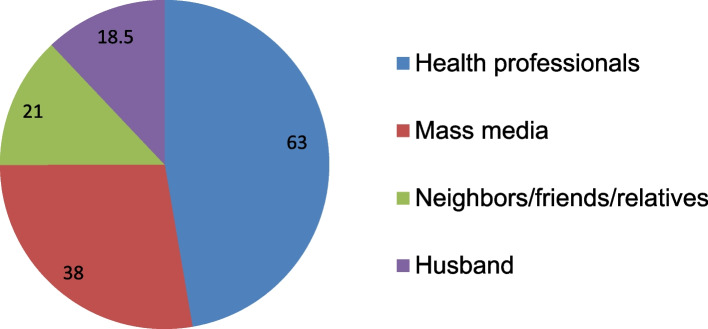
Source of information about PPIUCD for pregnant women attending ANC at Debre tabor town public health institutions, North West, Ethiopia, 2021

Of the total respondents, (56.1%) of women had awareness about PPIUCD. Among thus majority, (63%) of study participants, heard from health professionals (Figs. 1 and 2).

### Knowledge of pregnant women about post-partum intrauterine contraceptive device

This study indicated that more than one-third (36%; 95% CI: 32.4–39.6) of study participants had good knowledge about PPIUCD. The majority of 287 women (68.8%) responded that PPIUCD is inserted free of charge in Ethiopia. where only 31.9% of women responded that PPIUCD cannot cause cervical cancer (Table 4).

**Table 4:** attitude towards PPIUCD among pregnant women attending ANC at Debre tabor town public health institutions, Northwest, Ethiopia, 2021 (*n* = 417)

Variable	Category	Frequency	Number
Insertion of PPIUCD inside the uterus Leads to loss of privacy.	Strongly Agree	14	3.4
	Agree	84	20.1
	Neutral	104	24.9
	Disagree	146	35.0
	Strongly disagree	69	16.6
Using PPIUCD restrict normal activities	Strongly Agree	24	5.8
	Agree	55	13.2
	Neutral	53	12.7
	Disagree	193	46.3
	Strongly disagree	92	22
PPIUCD move through the body after insertion.	Strongly Agree	40	9.6
	Agree	93	22.3
	Neutral	93	22.3
	Disagree	100	24.0
	Strongly disagree	91	21.8
PPIUCD interfere with sexual intercourse	Strongly Agree	45	12.9
	Agree	106	24.8
	Neutral	87	20.1
	Disagree	81	19.9
	Strongly disagree	98	22.3
PPIUCD can harm a woman’s womb	Strongly Agree	65	15.6
	Agree	105	25.2
	Neutral	64	15.3
	Disagree	88	21.1
	Strongly disagree	95	22.8

### Attitude towards PPIUCD among pregnant women

This study showed that 48.7% (95% CI: 44.6–55.2) of women had a positive attitude towards PPIUCD, with a mean attitude score of 16.3 (SD 4.8). Thirty five percent of mothers disagreed that the insertion of an IUCD inside the uterus leads to a loss of privacy. Less than half (46.3%) of women disagreed that using IUCD restricts normal activities, and 24% of women disagreed that IUCD moves through the body after insertion (Table 5).

**Table 5:** Factors associated with knowledge about PPIUCD among pregnant women attending ANC at Debre tabor town public health institutions, Northwest, Ethiopia, 2021 (*n* = 417)

Variables	Knowledge about PPIUCD		COR(95%CI)	AOR(95%CI)	*P* value
	Good knowledge	Poor knowledge			
Educational status					
No education	**21**	**80**	**1**	**1**	
Primary (1–8)	**18**	**44**	**1.56(0.75–3.23)**	**0.92(0.39–2.16)**	0.693
Secondary (9–12)	**39**	**92**	**1.62(0.88–2.97)**	**0.96(0.47–1.95)**	0.532
College and above	**72**	**51**	5.38(2.95–9.80)**	**1.18(0.50–2.76)**	0.478
Occupational status					
Housewife	**39**	**144**	**1**	**1**	
Government employee	**77**	**39**	7.29(4.32–12.30)**	4.98(2.79–8.91)**	0.000
Private employee	**17**	**40**	**1.57(0.80–3.06)**	**1.39(0.66–2.91)**	0.383
Daily laborer	**8**	**22**	**1.34(0.56–3.25)**	**1.10(0.42–2.92)**	0.075
Farmer	**4**	**17**	**1.85(0.28–2.73)**	**0.87(0.52–6.59)**	0.971
Student	**5**	**5**	5.29 (1.02–13.40)*	3.69(1.29–21.80)*	0.027
Residence					
Ruler	**24**	**90**	**1**	**1**	
Urban	**126**	**177**	2.67(1.61–4.42)**	1.90(1.02–3.53)*	0.008
Ever discussed on PPIUCD with a health care provider					
No	**43**	**198**	**1**	**1**	
Yes	**107**	**69**	7.14(4.56–11.17)**	6.01(3.709.744**	0.000

AOR Adjusted odds ratio, COR Crude odds ratio, CI Confidence interval, 1 Reference ^**^P ≤ 0.001, ^*^P ≤ 0.05

### Factors associated with knowledge about PPIUCD among pregnant women

In binary logistic regression: educational status, occupation, residence, and ever had a discussion on PPIUCD with health care provider was found to be a *p*-value of < 0.2 and entered into the multivariable analysis. Finally, occupation, residence, and ever discussed of PPIUCD with a healthcare provider were significantly associated with knowledge about the use of PPIUCD in the multivariable analysis Table 6.

**Table 6:** Factors associated with attitude towards PPIUCD among pregnant women attending antenatal care at Debre tabor town public health institutions North West Ethiopia, 2021(*n* = 417)

Variables	Attitude towards PPIUCD		COR(95%CI)	AOR(95%CI)	*P* value
	Positive	Negative			
Educational status					
No education	24	77		1	
Primary	26	36	2.32 (1.17–4.58)	1.55 (.587–4.08)	0.378
Secondary	65	66	3.22(1.78–5.60)	3.16(1.41–7.31) *	0.005
College and above	88	35	8.07(4.42–14.74)	3.62(1.75–7.51) *	0.032
Occupational status					
Housewife	60	123	1	1	
Goven‟t employee	93	23	8.29(4.78–14.38)	2.76(1.11–6.81) *	0.028
Private employee	22	35	1.29(.70–2.39)	.621 (0.24–1.58)	0.319
Daily laborer	13	17	1.90 (.72–3.44)	1.57 (0.57–6.36)	0.299
Farmer	6	15	.82(.30–2.22)	.688 (0.17–2.80)	0.601
Student	9	1	32.10 (2.29–25.81)	18.45(3.22–44.79) **	0.003
Residence					
Rural	43	71	1	1	
Urban	160	143	1.85 (1.19–2.87)	.50(0.24–1.07)	0.312
Knowledge about PPIUCD					
Poor knowledge	70	197	1	1	
Good knowledge	133	17	22.02(12.4–39.1)	13.72(6.63–28.42) **	0.000
Ever discussed on PPIUCD with a health care provider					
No	78	163	1	1	
Yes	125	51	5.12 (3.36–7.82)	2.24(1.18–4.24) *	0,014
Number of ANC					
One	26	109	1	1	
Two	53	63	4.46 (2.01–6.19)	3.53(2.10–9.96) **	0.000
Three	57	25	9.56(5.06–18.05)	9.41(3.98–22.21) **	0.000
Four	67	15	24.20 (9.26–37.88)	18.73(9.34–62.67) **	0.000
Ever use modern contraceptive methods					
Yes	301	14	2.13(1.37–3.32)	1.01(0.513–2.00)	0.867
No	75	139	1	1	

AOR Adjusted odds ratio, COR Crude odds ratio, CI Confidence interval, 1 Reference ^**^P ≤ 0.001, ^*^P ≤ 0.05

The government-employed women were 5 times more likely to have good knowledge about PPIUCD than housewives (AOR = 4.98, 95%CI: 2.79–8.91). Furthermore, women who were students were 4 times more likely to have good knowledge about PPIUCD as compared to housewives (AOR = 3.69, 95%CI: 1.29–21.80).

Women who were from urban residences were 2 times more likely to have good knowledge about PPIUCD than women who were from rural residences (AOR = 1.90,95%CI: 1.02–3.53).

Women who discussed with health care providers about PPIUCD were 6 times more likely to have good knowledge about PPIUCD than their counterparts (AOR = 6.01, 95%CI: 3.70–.7.44).

### Factors associated with attitude of pregnant women towards of PPIUCD

In binary logistic regression: educational status, occupation, knowledge about PPIUCD, residence, Number of ANC, ever discussed on PPIUCD with the health care provider, and Ever use modern contraceptive methods was found to be a *p*-value of < 0.2 and entered to multivariable analysis. Finally, educational status, occupation, knowledge of PPIUCD, ever discussed of PPIUCD with a health care provider, and number of ANC were significantly associated with attitude towards the use of PPIUCD in the multivariable analysis.

Women who had attained secondary education were 3.2 times more likely to have a positive attitude towards PPIUCD than women who had no formal education (AOR = 3.16, 95%CI: 1.41–7.31). Moreover, women who had attended college and above education were 3.6 times more likely to have a positive attitude towards PPIUCD as compared to those who had no formal education (AOR = 3.62,95%CI:1.75–7.51).

The government-employed women were 2.8 times more likely to have a positive attitude towards PPIUCD than housewives (AOR = 2.76, 95CI:1.11–6.81). Furthermore, women who were students were 18 times more likely to have a positive attitude towards PPIUCD as compared to housewives (AOR = 18.45, 95%CI: 3.22–44.79).

Women who had good knowledge were 14 times more likely to have a positive attitude towards PPIUCD than women who had poor knowledge (AOR = 13.72, 95%CI: 6.63–28.42).

Women who discussed with health care providers about PPIUCD were 2.2 times more likely to have a positive attitude towards PPIUCD than their counterparts (AOR = 2.24, 95CI:1.18–4.24).

Women who have two ANC follow up were 4 times more likely to have a positive attitude towards PPIUCD than women who have only one ANC follow-up (AOR = 3.53,95%CI:2.10–9.96). Women who have three ANC follow up were 9.4 times more likely to have a positive attitude towards PPIUCD than women who have only ANC follow-ups (AOR = 9.41, 95%CI: 3.98–22.21). Furthermore, women who have four ANC follow up were 19 times more likely to have a positive attitude towards PPIUCD than women who have only one ANC follow-up (AOR = 18.73,95%CI:9.34–62.67).

## Discussion

This study was conducted to assess knowledge and attitude towards PPIUCD and associated factors among pregnant women attending ANC at Debre tabor town public health institutions Northwest, Ethiopia, 2021.

Knowledge about PPIUD was 36% % within the study area. This finding is in line with the findings of a study done in Northern India (36%) [22] and Nigeria (34.4%) [23]. However, the result of this study is lower than a study done in Ambo town, Ethiopia (48%) [18], Tanzania(86.7%) [24], and India (44.8%) [16]. The possible explanation for this difference from the studies conducted in Ambo town might be due to time differences and differences in sample size. Another reason might be due to the difference in socio-demographic characteristics: The majority of respondents had completed secondary education (36.9%) in Ambo town however; in this study, 24.7% of respondents have no formal education. The possible explanation for the difference in this study from Tanzania might be the difference in the study population: unlike this study studies done in Tanzania were among none pregnant women.

The possible reason for the difference in this study from India might be due to differences in socio-demographic characteristics: in the India study Majority (83.2%) of respondents were above primary education but in this study, only 60.9% of the respondents were above primary education.

The result of this study was higher than the results of studies done in India (4%) [25]. This difference might be due to the time difference. Furthermore, these differences might be due to differences in socio-demographic characteristics: in the India study only 19.34% of women completed higher education but in this study about 29.5% of the study, participants completed higher education. However, Different studies showed that better-schooled couples have a wider knowledge of contraceptive methods than others [26, 27].

Based on the finding of this study, employment was associated with knowledge about PPIUCD. Government-employed women were 5 times more likely to have good knowledge about PPIUCD than housewives. Furthermore, women who were students were 4 times more likely to have good knowledge about PPIUCD as compared to housewives. This finding was supported by the study conducted in Ethiopia [28]. This might be due to employed women might have access to media and the chance to get updated information from other staff members. In addition to this, Women who were students can access information from, the internet, read a newspaper, and their teachers and classmates.

Based on the finding of this study, the residence was associated with knowledge about PPIUCD. Women who were from urban residences were 2 times more likely to have good knowledge about PPIUCD than women who were from rural residences. This finding was supported by the study conducted in Ethiopia [28]. This might be due to women who were from urban residences can access information from different sources like newspapers, TV, and the internet.

Women who discussed with health care providers about PPIUCD were 6 times more likely to have good knowledge about PPIUCD than their counterparts. This finding was supported by the study conducted in India [16]. This might be due to women who ever discussed PPIUCD with health care providers might get clear information about PPIUCD.

Positive attitude towards PPIUCD was 48.7% in the study area. This finding is in line with the findings of a study done in Ambo town, Ethiopia (49.4%) [18]. The result of this study was higher than the study done in India (43.3%) [29], and in Tanzania(33.3%) [24]. The difference in this study from India might be due to the difference in sampling technique; a study conducted in India uses a non-probability (convenience) sampling technique. However, in this study, a probability (systematic random) sampling technique was used. In addition to this, the difference might be due to the difference in sample size: the study in India used an inadequate sample size (*n* = 180). This difference might be also due to differences in socio-economic and socio-demographic characteristics. In the India study, only 28.9% of study participants attained college and above education but in this study, about 30.7% of women attained college and above education. In addition, to this in the India study, only 5% of women were government employed but in this study, about 27.8% of women were government employed. However, studies showed that government-employed women had positive attitude than housewives [30].

The difference in this study from Tanzania might be due to the difference in the study population. Furthermore, this might be due to differences in socio-economic and socio-demographic characteristics. However, the result of this study is lower than a study done in the west Gojjam zone, Ethiopia (55.32%) [19] This difference might be due to differences in the study population and socio-demographic and socio-economic characteristics: in the west Gojjam zone, Ethiopia study about 31.2% of women were employed but in this study, only 27.8% of women were government employed. But, studies showed that government-employed women had positive attitude than housewives [30].

Women who had attained secondary education were 3.2 times more likely to have a positive attitude towards PPIUCD than women who had no formal education. Moreover, women who had attended college and above education were 3.6 times more likely to have a positive attitude towards PPIUCD as compared to those who had no formal education. This result was supported by the study done at Jemma Zone, Ethiopia [31] in Turkey [30] . The possible explanation might be due to educated women having more access to information from different sources like the internet, school, and newspaper. This might be also due to educated women can understand the message easily, and might have received lessons on contraceptive methods in the curriculum at school.

The government-employed women were 2.8 times more likely to have a positive attitude towards PPIUCD than housewives. Furthermore, women who were students were 18 times more likely to have a positive attitude towards PPIUCD as compared to housewives. This result was supported by the study done in Turkey [30] .This might be due to employed and student women might have access to media and the chance to get updated information from other staff members and classmates. This might be also women who were students might get information from their teachers and might have received lessons on contraceptive methods in the curriculum at school.

Women who had good knowledge were 14 times more likely to have a positive attitude toward PPIUCD than women who had poor knowledge. This result was supported by the study done in Turkey [30]. This might be due to women who have good knowledge can differentiate the different myths and misconceptions speech by the community.

Women who discussed with health care providers about PPIUCD were 2.2 times more likely to have a positive attitude towards PPIUCD than their counterparts. This finding was supported by the study done in.

Turkey [32] his might be due to discussion with a health care provider that can remove rumors and misconceptions about PPIUCD.

Women who have two ANC follow up were 3 times more likely to have a positive attitude towards PPIUCD than women who have only one ANC follow-up. Women who have three ANC follow up were 9.4 times more likely to have a positive attitude towards the use of PPIUCD than women who have only one ANC follow-up. Furthermore, women who have four ANC follow up were 19 times more likely to have a positive attitude towards PPIUCD than women who have only one ANC follow-up. This study is supported by a study done in turkey [30]. This might be due to women who have more contact with health workers can access more information about reliable contraceptive methods and can remove rumors and misconceptions about PPIUCD.

## Limitations

Because of time and logistic constraints, the study used only quantitative approach; as a result, women’s feeling was not assessed deeply.

Recall bias and social desirability bias might be introduced. Because of the study was used cross sectional study design selection bias might be introduced, Chicken egg dilemma (which one occurred first is not known), Generalizability limited by sampled population and population definition, It doesn’t show the temporal relationship and It evaluates prevalence rather than incidence.

## Conclusion and recommendation

In this study, the overall score of knowledge and attitude was found to be low. This might be mainly attributed to the low achievement of education, living in a rural area, and not discussing PPIUCD with a health care provider. Government employees, being students, living in an urban area, and ever discussed on PPIUCD with a health care provider were associated with knowledge of women about PPIUCD. Had completing secondary education, government employee, being a student, having good knowledge, ever discussed PPIUCD with a health care provider, and attending two and more antenatal care visits were associated with attitude towards PPIUCD.

Therefore, due attention should be given to enhancing the educational level of women, encouraging women to have better academic involvement and achievement, to have ANC follow up and effective IUCD counseling should be given during antenatal care to increase knowledge and create a good attitude about PPIUCD.

Continuous education and awareness creation session should be arranged at the community level to increase women’s knowledge and to create a positive attitude about the PPIUCD.

## Supplementary Information

**Additional file 1.** Annex 1. Consent Form. Annex 3. English Version Questionnaires.

## Data Availability

Additional data and materials can be available through a request to the corresponding author.

## Abbreviations

ANC: Antenatal Care
AOR: Adjusted Odds Ratio
CI: Confidence Interval
COR: Crude Odds Ratio
EDHS: Ethiopian Demographic and Health Survey
FP: Family Planning
IUCD: Intrauterine Contraceptive Device
PPIUCD: Postpartum Intrauterine Contraceptive Device
SDG: Sustainable Development Goals
SPSS: Statistical Package for Social Science
OR: Odis Ratio
