# Supplementary material for: Factors associated with knowledge of the postpartum intrauterine contraceptive device and attitude towards its use among women attending antenatal care at Debre Tabor town, Northwest Ethiopia, 2021: a cross-sectional study
Source: Contracept Reprod Med. 2023 Jan 15;8:7. doi: 10.1186/s40834-022-00202-y (PMC9840842; doi:10.1186/s40834-022-00202-y)
Supplement: Supplementary file 1 — Additional file 1. Annex 1. Consent Form. Annex 3. English Version Questionnaires. [file 40834_2022_202_MOESM1_ESM.docx]

# 11. ANNEXE

Annex 1: Consent Form

My name is ________________ I am one of the members of the research team conducted by Fillorenes Ayalew, Wassie Yaze, Abeba Belay and Besefat Berihun the objective of this research is *to assess the* magnitude of knowledge and attitude towards postpartum intrauterine contraceptive device and associated factors among pregnant women attending antenatal care at Debretabor town public health institutions, North West Ethiopia, It is my pleasure to notify you that you have been identified to participate in this study. I am going to ask you few questions which are very important and related to PPIUCD. Your name will not be written in this form and the information you will give to us is kept confidential. Your inclusion in the study is voluntary and you are free to withdraw from the study if you are not voluntary to participate. However, your willingness to answer all of the questions would important to the organization and the community. It doesn’t take more than 20 minutes.

Would you participate in the study?

Yes ___________

No __________

If the answer is yes, thanks! Conduct

If the answer is No, thanks! Transfer to the next respondent

Signature of participant ____________

Name and signature of the data collector who sought the consent ______________

Date of interview________________________

Name and signature of the supervisor ___________________

Date _________

Annex 3: English Version Questionnaires

Part I: socio-demographic characteristics of study participants and their partners

| s/no | Questions | | | Answers/choices | Remark | | |
| --- | --- | --- | --- | --- | --- | --- | --- |
| 101 | Age | | | --------- complete years |  | | |
| 102 | Religion | | | 1.Orthodox  2.Muslim  3.Protestant  4.Catholic  5.other specify-------------- |  | | |
| 103 | Where is your residence? | | | 1.Rural  2.Urban |  | | |
| 104 | Educational status | | | 1. No education  2. Primary (1-8)  3. Secondary (9-12)  4 .College and above |  | | |
| 105 | Occupational status | | | 1. Housewife  2. Government employee  3. Private employee  4. Daily laborer  5 .Farmer  6. Student  7 .Other (specify --------- |  | | |
| 106 | Ethnicity | | | 1.Amhara  2.Tigre  3.Oromo  4.Others specify------------ |  | | |
| 107 | Current marital status | | | 1.Single  2.Married  3.Divorced  4.Widowed  5.Other specify------- | If your answer is not 2 skip to Q no 111 | | |
| 108 | Husband educational status | | | 1. No education  2. Primary (1-8)  3. Secondary (9-12)  4. College and above |  | | |
| 109 | Husband occupational status | | | 1.Farmer  2.Daily laborer  3.Merchant  4.Government employee  5.Private employee)  5 .Student  6. Other (specify------- |  | | |
| 110 | How much is your family's average monthly income? | | | ----------------------in(ETB) |  | | |
| **Part II: obstetrics/reproductive related questions** | | | | | | | |
| 201 | Have you ever give birth? | | 1.Yes  2.No | | | If your answer for Q 202 is No skip to Q. 207 | |
| 203 | How many birth you give | | -------birth/s | | |  | |
| 205 | How many total numbers of deliveries you had including stillbirths/neonatal death*s)* | | ------------ delivery/deliveries | | |  | |
| 207 | Do you want to have a child within two years of delivery? | | 1.Yes  2.No | | | If your answer for Q207 is Yes skip to Q no 209 | |
| 208 | If your answer to question number 207 is No, why? | | 1.To space  2.To limit  3.Other(specify)__________ | | |  | |
| 209 | How many children do you want to have in your life? | | --------- child/children | | |  | |
| 210 | Do you ever discuss with your partner on family planning method | | 1.Yes  2. No | | |  | |
| 211 | Have you ever discussing about PPIUCD with a health care provider? | | 1.Yes  2.No | | |  | |
| 212 | Who decides/will decide on the no of children you want to have?? | | 1.My husband/partner  2. Myself  3. Both of us  4.God  5. Others (specify ---------- | | |  | |
| 213 | Who is a responsible person for health care decisions? | | 1. My husband/partner  2. Myself  3. Both of us  4. Others (specify-------------- | | |  | |
| 214 | Have you ever used a modern contraceptive method? | | 1.Yes  2.No | | | If your answer is No for Q no 214 skip to Q no 218 | |
| 215 | *If, your answer is yes for question number 214 which method you used?(more than one answer is possible)* | | *1.Pills*  *2.Injectable*  *3.Implant*  *4.IUCD*  *5.Condom*  *6.Other(specify)_________* | | |  | |
| 216 | *For how many years did you use?* | | *---------------completed years* | | |  | |
| 217 | Have you ever shifted from one contraceptive method to another? | | 1.Yes  2.No | | |  | |
| 218 | Who decides on the use of family planning? | | 1.Myself  2.My husband/partner  3.Both of us  4.Health care provider  5. Others (specify)……… | | |  | |
| **Part III: Awareness and knowledge about PPIUCD of the participants**  **Awareness about PPIUCD of the participants** | | | | | |  | |
| 301 | Have you ever heard IUCD can be inserted immediately after delivery? | | 1.Yes  2.No | | | If your answer is No for Q 301 skip to Q 303 | |
| 302 | If your answer is yes, for question number 301 from whom/where did you hear? ( more than one answer is possible) | | 1.Neighbors/friends/relatives  2.Healthprofessionals/worker  3.Mass media (TV, radio)  4.Husband/Partner  5.Others (specify) ______ | | |  | |
| **Participants knowledge about PPIUCD** | | | | | | | |
| 303 | PPIUD can prevent unwanted pregnancies for more than 10 years | 1.Yes  2.No | | |  | | |
| 304 | PPIUCD has no risk of getting sexually transmitted infections | 1.Yes  2.No | | |  | | |
| 305 | PPIUCD has no interference with sexual intercourse | 1.Yes  2.No | | |  | | |
| 306 | PPIUCD is immediately reversible | 1.Yes  2.No | | |  | | |
| 307 | PPIUCD cannot cause cervical cancer. | 1.Yes  2.No | | |  | | |
| 308 | PPIUD can be used by breastfeeding mothers | 1.Yes  2.No | | |  | | |
| 309 | PPIUD may cause changes in bleeding pattern | 1.yes  2.no | | |  | | |
| 310 | PPIUCD can be used by HIV positive patients doing well on treatment | 1.Yes  2.No | | |  | | |
| 311 | PPIUD is inserted free of charge in Ethiopia | 1.Yes  2.No | | |  | | |
| 312 | PPIUD can be removed at any time you wish | 1.Yes  2.No | | |  | | |
| **Part IV: Pregnant women’s attitude to use PPIUCD** | | | | | | | |
| **Pregnant women’s attitude towards PPIUCD** | | | | | | | |
| 401 | Insertion of PPIUCD inside the uterus Leads to loss of privacy. | | 1. Strongly disagree  2. Disagree  3. Neutral  4. Agree  5.Strongly agree | | | |  |
| 402 | Using PPIUCD restricts normal activities. | | 1. Strongly disagree  2. Disagree  3. Neutral  4. Agree  5. Strongly agree | | | |  |
| 403 | PPIUCD move through the body after insertion. | | 1. Strongly disagree  2. Disagree  3. Neutral  4. Agree  5. Strongly agree | | | |  |
| 404 | PPIUCD interfere with sexual intercourse | | 1. Strongly disagree  2. Disagree  3. Neutral  4. Agree  5.Strongly agree | | | |  |
| 405 | PPIUCD can harm a woman’s womb. | | 1. Strongly disagree  2. Disagree  3. Neutral  4. Agree  5. Strongly agree | | | |  |
